# Progress in Otology

**Published:** 1895-11-23

**Authors:** 


### Progress in Otology.

(*Continued from page* 120.)

**A New Form of Rotatory Vertigo.**—Professor Guye[3] has described a case of Menière's disease in which the rotatory sensations differ from those already known. Instead of feeling as though he was turning on a pivot in the vertical axis, in a direction towards the affected side, with a tendency to turn involuntarily in the same direction, the patient thought he saw objects turning before him, and in the direction of the hands of a clock, after which they appeared to turn away to the right. The other symptoms were typical. Guye seeks to explain the various rotatory sensations by reference to the anatomical position ef the semi-circular canals. The common form (rotation around a vertical axis) might be accounted for by supposing the existence of some irritation in the ampullæ of the horizontal canal. A slight rotatory sensation in the superior canal would give rise to a feeling of falling forwards or backwards, but around an axis, which instead of being exactly transverse is inclined to the right or left, according to the side affected. An affection of the posterior canal would give rise to a sensation of objects rotating in front of the patient, like the hands of a clock, the disc of which is not in front, but to the right or left of the patient, as the case may be.

**Discussion on Nerve Deafness at the British Medical Meeting**, 1895.—Dundas Grant,[4] in introducing this, classified the disease into (1) labyrinthine, including (i) labyrinth alone, due to various causes, *e.g.*, the action of sound, circulatory disturbances, infectious disease, traumatism, &c.; (ii) labyrinth, secondary to middle-ear inflammation, *e g.*, chronic catarrhal (including typical Menière's disease), suppurative, &c.; (2) diseases of the auditory nerve and cerebral auditory centres, *e.g.*, neuro-labyrinthitis following suppurative meningitis, cerebral syphilis, lesions of the cerebral centres, hysteria, &c., &c. In treating the first division depletion was first to be thought of; syphilitic cases were benefited by profuse perspiration, hence pilocarpine was always to be tried in the first instance. Deafness from noise should be prevented by stopping the ears with wool. Galvanism was serviceable, especially for the tinnitus, the positive pole being applied to the nape of the neck, and the negative to the tragus or mastoid; four to five milliampères might be employed. Pilocarpine should be injected in labyrinthitis following mumps, and also in leukæmia and infectious diseases. A case of almost complete recovery in a case following scarlet fever has been reported in the "Arch. of Otol." vol. iv. Sedatives, as the bromides; depressants, as colchicum and the iodide; tonics, as strychnine and quinine, and counter-irritants, were all indicated in suitable cases. The discussion turned chiefly upon prophylaxis; the importance of treating the middle ear when this element entered into the case; the value of pilocarpine (which had been found very disappointing by most of the speakers); and the benefit which had so far been obtained from mercury and the iodide in secondary, tertiary, or inherited syphilis. Dr. Law had succeeded in clearing up all the symptoms in two cases of secondary syphilis with the usual anti-specific remedies, but the deafness remained unaffected. In the tertiary and inherited form he had not met with any better success.

**Aspergillus Flavescens in the Auditory Canal.**—Pomeroy,[5] of New York, reports a case of this disease, in the treatment of which he found that strong solutions of nitrate of silver, and even alcohol, were insufficient to destroy the fungus. Better success followed the use of liq. sodæ. chlor. full strength, which entirely destroyed the aspergillus in three daily instillations continued for two days. Burnett finds that a powder composed of boric acid 16 parts and chinoline salycilate one part is quite successful as an insufflation in one application.

**Rupture of the Membrana Tympani from Violence**[6] is not uncommon as the result of a box on the ears, or a fall on the side of the head, the sudden compression of the air in the meatus being sufficient to burst the drum. A case is reported from San Francisco, in which a person inflicted a slap on his own ear to dislodge water which entered whilst bathing. The water was forced from the canal into the middle ear, and set up violent otitis media, with pain and serous discharge. In such cases a pledget of iodoform gauze in the meatus is sufficient treatment.

**A New Mode of Treating Furuncle of the Meatus**[7] consists in inserting an india-rubber tube, which is intended to act both as a drain for the pus and as a dilator for maintaining the patency of the canal. It is said that the tube relieves the pain of the affection when incisions are ineffectual, and also enables thorough cleansing to be carried out in the meantime.

**Salycilic Acid Collodion in Otitis Externa.**—Dunn[8] recommends one drachm of salycilic acid in an ounce of collodion, to be applied once daily with a camel's hair brush. This application is said to be most useful for affections due to aspergillus when the membrane is not involved. It also relieves the itching in dry scaly conditions of the external meatus. There is some pain after the application, but it soon passes off.

MacCuen Smith[9] recommends camphor-phenol for cutting short incipient furunculosis, and summarises thus the treatment of this disease: (1) As antiphlogistic measures, leeching or blistering in front of the tragus, and hot antiseptic irrigation when this is indicated. Avoid poultices. (2) As a local application, cleanse the canal with alcohol and insert an ample tampon of cotton wool saturated with camphor phenol, renewing this every twenty-four hours or oftener if required. (Antiseptic and analgesic.) (3) As constitutional remedies, tonics and alteratives, especially arsenic in Fowler's solution, given until the physiological action is obtained. (4) In well-advanced and chronic cases, and when pus has formed, free incision through the furuncle, dividing the periosteum down to the bone.

**Tuberculous Disease of the Middle Ear.**[10]—Milligan has conducted a most original series of experiments, which prove that tubercle oftener attacks the middle ear than is commonly believed, painless perforation of the membrane, especially when accompanied by facial paralysis, usually indicating tuberculous mischief. He operated upon ten cases of mastoid suppuration in children, and inoculated guinea pigs with scrapings from the diseased bone. In eight of the ten cases tuberculous infection followed, and upon dissection the abdominal and pelvic glands were found to show distinct evidence of tuberculous infiltration.

**Cerebral Complications in Relation to Middle Ear Disease.**—Dr. Macewen,[11] in opening a discussion on this subject at the recent British Medical Association meeting in London, dwelt largely upon tubercle of the middle ear, remarking that this had not received the attention it deserved. Spreading slowly, and with little or no pain or discharge, it sometimes reached an advanced stage without perforation. Tuberculous products formed excellent culture media for pyogenic bacteria, and the latter easily found their way into the softened bones. Tubercle bacilli also invaded the blood stream, *viâ* the lateral sinus and internal jugular. In operating it was essential to open the mastoid and expose and eradicate the diseased foci. He recommended the dental burr made by the American Dental Company as preferable to all other instruments for mastoid operations. There was always a probability of tuberculous disease being present in suppurating bone, but a general tuberculous infection was preventable when the disease was limited to the mastoid. When the disease had spread to the petrous bone it was difficult to eradicate, but the infected cavities must be opened up, and the walls lined with healthy skin to allow free exit for the discharge. Mr. Ballance remarked upon the serious lesions in distant parts, apart from cranial complications, that are apt to follow tuberculosis of the middle ear. Children suffering from hip and spinal disease often had a history of aural discharge.

**Inflammation of the Middle Ear of Infants.**—Hartmann and Rossell[12] give the following results of their investigations on this subject, made in the Berlin Institute of Infectious Diseases: (1) Post-mortem and ante-mortem examinations in this institution show that 75 per cent. of the children suffer from inflammation of the middle ear; (2) inflammation of the middle ear in infants can nearly always be detected by an otoscopic examination; (3) the general symptoms of otitis media are restlessness, elevated temperature, and loss of weight: sometimes these symptoms are not present; (4) very often the symptoms of otitis media are connected with broncho-pneumonic processes, probably both due to aspiration at birth; (5) death can result in cases of otitis media from slow atrophy or from an extension of the micro-organisms into the cranial cavity or into the blood; (6) treatment must correspond with the various conditions existing.

**Treatment of Acute Otitis Media.**—Pes and Gradenigo[13] conclude from bacteriological researches that, as a rule, primary and secondary infections of the middle ear arise from the naso-pharynx, and that secondary infections are very rarely traceable from the meatus through a perforation in the membrana tympani. They hold (1) that there is an absence of staphylococcus pyogenes albus from the pus in the tympanic cavity, nose, and Eustachian tube; (2) it is present in prepared cotton wool; (3) it appears in the pus of the middle-ear after treatment.

Lermoyer and Helme[14] do not accept these conclusions. They point out (1) that pyogenic staphylococci are rare at the commencement of a suppurative otitis media, but become of extreme frequence as the suppuration proceeds; (2) that their slow approach is the result of secondary infection; (3) that all experience is in favour of the view that they obtain access *viâ* the external auditory meatus; (4) that staphylococcus pyogenes is pretty frequently found in cotton wool prepared in the usual way, except that in practice at Turin; and (5) that the most simple mode of sterilisation of the wool, in order to protect the meatus from inoculation, is saturation in boric alcohol and momentary suspension in the flame of a spirit lamp. This method prevents to some extent an acute purulent otitis media under treatment from becoming chronic. An earlier paper by Pes and Gradenigo[15] on "The Rational Treatment of Acute Otitis Media," is full of valuable hints to the practitioner. The following formula is recommended as an instillation to avert an attack of acute otitis media: ℞. Carbolic acid, ¾ to 1 gramme; chloride of sodium, 4 grammes; boiled distilled water, 50 grammes. This is a powerful antiseptic and analgesic; glycerine is omitted because, while neutralising the caustic action of the phenol, it also detracts from the effectiveness of the remedy. The chloride prevents the maceration of the epidermis of the meatus and membrane that soon follows upon repeated instillations. If the mischief is not arrested by this treatment, combined with rest in bed, dieting, gargling, nasal irrigations, and such like adjuvants, every means must be employed to assist nature in ridding the inflamed drum cavity of the pathogenic micro-organisms. When, therefore, rupture does not occur spontaneously, paracentesis must be performed, with the following precautions: The pinna and auditory meatus are washed with a 1:1,000 perchloride of mercury solution, and local anæsthesia is procured by means of a few drops of a 10 per cent. cocaine solution, to which has been added 1 per cent. of carbolic acid. After the operation every precaution must be taken to hinder the entrance of fresh infective germs into the drum cavity. Politzerisations, syringings, &c., are therefore to be avoided; even absorbent cotton twisted on the holder by the cleanest fingers is charged with staphylococci, and may prove a source of secondary infection. The best application, after careful removal of pus and blood from the meatus, is a fine pledget of iodoform gauze, introduced short of touching the membrane with the aid of speculum and forceps. The external end of the pledget is to be covered with a few layers of the same material; this dressing must be renewed once or twice daily, according to the amount of pus escaping.

[3] B.M. Assoc. meeting, rep. in Lancet, Aug. 24, 1895. [4] Ibid. [5] Am. Jnal. of Med. Sci., June, 1895. [6] Ibid. [7] Arch. Internat. de Lar., April, 1895. [8] Arch. of Otol., April, 1895. [9] Therap. Gazette, April, 1895. [10] B.M. Assoc. meeting, 1895, rep. in Lancet, Aug. 24. [11] Ibid. [12] Am. Jnal. of Med. Sci., June, 1895. [13] Ann. des Mal. de l'Oreille. [14] Ibid. [15] Amer. Jnal of Med. Soi., June, 1895.

